# One‐Step Thermochemical Conversion of Biomass Waste into Superhydrophobic Carbon Material by Catalytic Pyrolysis

**DOI:** 10.1002/gch2.201900085

**Published:** 2020-02-20

**Authors:** De‐Chang Li, Wan‐Fei Xu, Hui‐Yuan Cheng, Kun‐Fang Xi, Bu‐De Xu, Hong Jiang

**Affiliations:** ^1^ CAS Key Laboratory of Urban Pollutants Conversion Department of Applied Chemistry University of Science and Technology of China Hefei 230026 China

**Keywords:** biomass, catalytic pyrolysis, FeCl_3_, superhydrophobic carbon

## Abstract

Preparation of superhydrophobic carbon materials from lignocellulosic biomass waste via one‐step carbonization is very difficult due to the existences of polar functional groups and ashes, which are extremely hydrophilic. Herein, superhydrophobic carbon materials can be facilely synthesized by catalytic pyrolysis of biomass waste using FeCl_3_ as catalyst. The results show that the surface energy of lignin‐derived char (Char_L_) is significantly reduced to 19.25 mN m^−1^ from 73.29 mN m^−1^, and the water contact angle increased from 0 to 151.5°, by interaction with FeCl_3_. Multiple characterizations and control experiments demonstrate that FeCl_3_ can catalyze the pyrolytic volatiles to form a rough graphite and diamond‐like carbon layer that isolates the polar functional groups and ashes on Char_L_, contributing to the superhydrophobicity of the Char_L_. The one‐step catalytic pyrolysis is able to convert different natural biomass waste (e.g., lignin, cellulose, sawdust, rice husk, maize straw, and pomelo peel) into superhydrophobic carbon materials. This study contributes new information related to the interfacial chemistry during the sustainable utilization of biomass waste.

## Graphical abstract

A facile method of converting biomass waste into superhydrophobic carbon materials is proposed by catalytic fast pyrolysis. The mechanism is attributed to the capping of the carbon deposition on the char coupled with the increased roughness, catalyzed by FeCl_3_. The superhydrophobic biochar exhibits outstanding stability in harsh environments and excellent performance in oil/water separation and self‐cleaning surfaces.

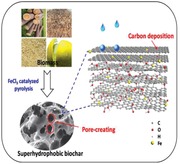

## 1 Introduction

Inspired by natural superwetting phenomena, materials with superhydrophobicity have been extensively explored due to their intriguing potential applications, such as water/oil separation,^[^ 1 ^]^ self‐cleaning,^[^ 2 ^]^ anti‐icing,^[^ 3 ^]^ and others.^[^ 4 ^]^ Previously, superhydrophobic carbon‐based materials, especially graphene and carbon nanotubes, have attracted significant attention arising from their low toxicity, outstanding physicochemical properties, and good chemical stability in the harsh environment.^[^ 5 ^]^ A general rule for fabricating superhydrophobic materials has been presented as combining a rough surface structure with low surface energy.^[^ 6 ^]^ Aiming to achieve excellent hydrophobicity for carbon‐based materials, high temperatures (above 1000 °C) were traditionally adopted to remove the surface polar groups,^[^ 6, 7 ^]^ generating a graphite‐like carbon structure with a low surface energy. The preparation processes for hydrophobic carbon‐based materials are complicated and costly, which greatly limits their large‐scale industrial applications.

Biomass waste, such as rice husk, sawdust, and straw, are renewable and readily available resources that have been used for the production of renewable energy and carbonaceous materials.^[^ 8 ^]^ However, although several publications have reported the preparation of hydrophobic carbon materials by pyrolysis of pure cellulose or mainly cellulosic biomass (Table S1, Supporting Information), there is currently no report on the preparation of superhydrophobic biochar materials using natural lignocellulosic biomass waste.^[^ 9 ^]^ The main challenge lies in the extremely complex and diverse composition of biomass, which is replete with polar atoms such as O, N, P, S, and metals. These heteroatoms can be incorporated with benzene‐rich structures, especially lignin, and are difficult to completely remove under the traditional pyrolysis conditions, ultimately forming polar sites and endowing the biochar with a hydrophilic or weakly hydrophobic property.^[^ 10 ^]^ Based on the published reports, after pyrolysis at 900 °C, the oxygen content of lignin‐derived biochar is still close to 10%, and most of the metals remain in the biochar.^[^ 11 ^]^ In addition, the hydrophilic ashes are ubiquitously distributed in natural biomass and impossible to be completely removed considering the cost.

Even if the polar sites are mostly removed, the low surface roughness of biochar makes it less superhydrophobic due to the lack of regular micron‐level fluctuation of the surface.^[^ 6b ^]^ Although some postprocessing methods, such as chemical vapor deposition,^[^ 5, 12 ^]^ dip‐coating,^[^ 13 ^]^ spraying,^[^ 14 ^]^ chemical modification,^[^ 15 ^]^ and casting^[^ 16 ^]^ can be employed to enhance the hydrophobic property of biochar, they are usually involved complex processes with high cost. Additionally, traditional methods for superhydrophobic modification via physical coverage or chemical grafting are prone to instability, especially under thermal stress and outdoor weatherability, leading to difficulty in meeting practical requirements.

Herein, we devised a universally applicable and facile method for preparing superhydrophobic biochar material derived from conventional lignocellulosic biomass waste. Given that Fe‐based catalysts have been widely used in the catalysis of carbon deposition for preparing carbon materials,^[^ 17 ^]^ and the chlorides can greatly improve the porosity of biochar,^[^ 18 ^]^ FeCl_3_ was chosen as a catalyst. The organic volatile matters produced from biomass decomposition were catalytically deposited to form a low‐polarity graphite layer on the surface of biochar (solid product of biomass pyrolysis). The in situ formed carbon layer would cover the polar sites on the biochar, enhancing the hydrophobic characteristics. Meanwhile, the surface roughness of biochar was increased due to the emergence of a large number of pores during the catalytic pyrolysis process, magnifying the hydrophobic property to superhydrophobility. Lignin was chosen as the study subject in the experiment process considering its hydrophilic nature, and the universality of this method was verified using typical lignocellulosic biomass feedstocks (i.e., sawdust, rice husk, maize straw, and pomelo peel).

## 2 Results and Discussions

### 2.1 Preparation of Superhydrophobic Biochar

**Figure 1:**
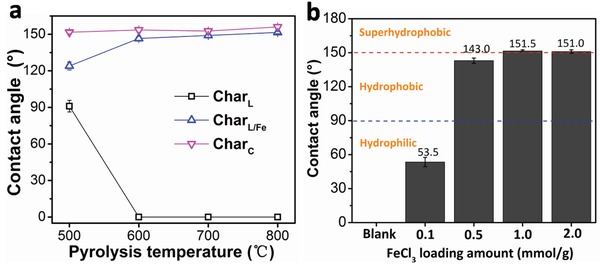
a) Water contact angles of Char_L_, Char_L‐ash_, Char_L/Fe_, and Char_C_ prepared at different temperatures; b) Water contact angles of Char_L/Fe_ with different FeCl_3_ loading amounts.

Lignin and cellulose, which are the main components of lignocellulosic biomass,^[^ 10 ^]^ were chosen as the model compounds to pyrolyze obtaining Char_L_ and Char_C_, respectively. As shown in **Figure** **1**a and Figure S1, Supporting Information, all the water contact angles of Char_C_ exceeded 150° and change slightly in the range of 500–800 °C, showing the superhydrophobicity. The Char_L_ pyrolyzed at 500 °C exhibited weak hydrophobicity with contact angle of 91.0°, while got superhydrophilic when pyrolyzed at 600–800 °C. This phenomenon may be caused by the change of ash content in Char_L_. As the pyrolysis temperature increased from 500 to 800 °C, the ash content was concentrated from 5.93 to 9.88% (Table S2, Supporting Information), which made the biochar more hydrophilic.

To achieve the superhydrophobicity of Char_L_, the lignin was preloaded with FeCl_3_ and pyrolyzed under the same conditions. Under the catalysis of FeCl_3_, great changes took place for the wettability of Char_L/Fe_ compared with that of Char_L_. The contact angle of Char_L/Fe_ pyrolyzed at 500°C was 124°, 33° larger than that of Char_L_. More significantly, the contact angle of Char_L/Fe_ got to 146.5° when pyrolyzed at 600 °C by comparison with the ≈0° of Char_L_, and continued to increase slowly as the temperature rose (Tables S3 and S4, Supporting Information). When pyrolyzed at 800 °C, the Char_L/Fe_ became superhydrophobic with a water contact angle of 151.5°.

The effect of FeCl_3_ dosage on the wettability of the Char_L_ obtained at 800 °C was investigated. It can be seen from Figure 1b that the Char_L_ is hydrophilic with a water contact angle of 53.5° when the FeCl_3_ dosage is 0.1 mmol g^−1^, and the wettability becomes hydrophobic with increasing FeCl_3_ dosage. The largest water contact angle appears at the FeCl_3_ dosage of 1 mmol g^−1^, with no further improvement at 2 mmol g^−1^. This phenomenon may be because of the fact that when the loading of FeCl_3_ is low, the hydrophobic layer produced by organic volatile deposition cannot achieve complete coverage on the surface of char; as the loading reaches a certain amount, the distribution of catalyst can cover all the surface areas, and the excessive FeCl_3_ loading will give no more contribution. In the following paragraphs, the FeCl_3_ dosage is set as 1 mmol g^−1^ without special explanation.

### 2.2 Exploring the Mechanism Relating to Enhancement of Hydrophobicity

The solid surface energy directly influences its wetting property toward a certain liquid. Usually, a solid sample with smaller surface energy will exhibit greater hydrophobicity. The surface energies of biochar samples were measured and calculated according to the Owens−Wendt method.^[^ 19 ^]^ The surface energy of Char_L/Fe_ is 19.25 mN m^−1^, much lower than that of pristine Char_L_ (73.29 mN m^−1^). Table S5, Supporting Information, shows that the decrease in the polar force portion γ_s_^p^ is much larger than that in the dispersion forces γ_s_^d^, implying the decrease in surface energy is mainly due to the decrease in the supply of polar force. In the biochar material, the polar force is attributable to the existences of metals and polar functional groups on the surface.

XPS can be used to determine the atomic composition on material surface, and its detection depth is usually only several nanometers. While elemental analysis and AAS measures the elemental content in the whole sample matrix. Based on the results shown in **Table** **1**, the O/C value of Char_L_ measured with an elemental analyzer is 0.1181, which is close to that determined by the XPS (0.1182), indicating good homogeneity between sample surface and matrix for Char_L_. By contrast, the O/C value of Char_L/Fe_ determined by XPS is 0.0735, only about half of that determined by elemental analyzer (0.1395), and lower than that of Char_L_ (0.1182). In other words, the surface and bulk composition of Char_L/Fe_ is heterogeneous. The oxygen content on the surface of Char_L/Fe_ is lower than that in the matrix, and also lower than that on the surface of Char_L_. This implies that the superhydrophobic modification may result from the reduction of oxygen‐containing functional groups on the char surface by the interaction of FeCl_3_. However, no reports are found that FeCl_3_ can catalytically reduce the oxygen‐containing groups. Thus, it is reasonable that a new coating may be formed by the interaction of FeCl_3_ during pyrolysis.

**Table 1:** Elemental analysis results and atomic compositions from XPS results

Sample	Elemental analysis [wt%]						XPS result [at%]			Atomic ratio (bulkb))		Atomic ratio (surfacec))	
	C	H	Oa)	Na	Fe	S	C	O	Fe	O/C	Fe/C	O/C	Fe/C
Lignin	59.73	5.63	24.33	9.38	—	0.93	—	—	—	0.3055	—	—	—
FeCl_3_/lignin	—	—	—	—	—	—	75.53	19.71	1.58	—	—	0.2609	0.0209
Char_L_	79.20	1.18	12.47	6.07	—	1.08	89.2	10.54	—	0.1181	—	0.1182	—
Char_L/Fe_	72.11	1.49	13.41	2.50	9.57	0.92	92.56	6.8	0.44	0.1395	0.0285	0.0735	0.0048

a) The O content was calculated by difference

b) The atomic ratio of O/C or Fe/C in the bulk sample was calculated based on the elemental analysis results

c) The atomic ratio of O/C or Fe/C on the sample surface was calculated based on the XPS results.

The XPS spectra show that the surface Fe/C value of Char_L/Fe_ (0.0048) is much lower than that determined by the elemental analyzer (0.0285), suggesting the formation of carbon layer on the surface of Char_L/Fe_. The qualitative difference between the surface and matrix composition strongly demonstrates that the surface of Char_L/Fe_ is a new phase formed during the fast pyrolysis. On the basis of the above results, it can be concluded that with the effect of FeCl_3_, a new carbon layer with few polar sites was generated on the surface of Char_L_, changing the hydrophilic Char_L_ to hydrophobic and even superhydrophobic Char_L/Fe_.

**Figure 2:**
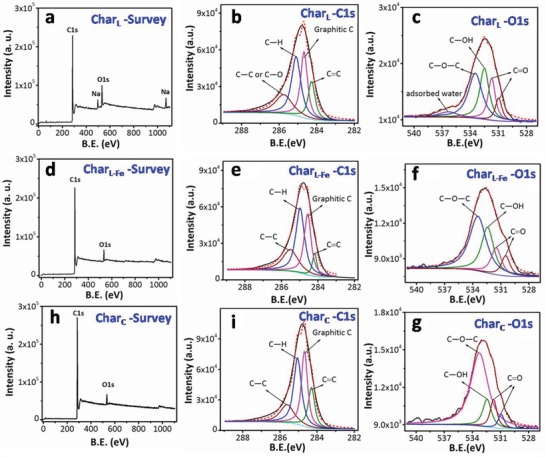
a) XPS survey, C 1s, O 1s spectra of a–c) Char_L_, d–f) Char_L/Fe_, and g–i) Char_C_, respectively.

The chemical states of C and O in the new coverage on Char_L/Fe_ were determined with XPS spectra (**Figure** **2** and Table S6, Supporting Information). In the C 1s spectra of Char_L/Fe_, the peaks at 284.18, 284.57, 284.98, and 285.51 eV can be attributed to C=C, graphitic C, C—H and C—C, respectively.^[^ 20 ^]^ The O 1s spectrum of Char_L/Fe_ contains four peaks at 530.48, 531.50, 532.42, and 533.45 eV, assigned to the C=O in quinines, C=O in ketones or aldehydes, C—OH and C—O—C, respectively.^[^ 21 ^]^ Compared with the O in Char_L_, O atoms in Char_L/Fe_ were more embedded into the carbon framework (C—O—C) and exhibited less polar groups, contributing to the outstanding hydrophobic property. Notably, a peak attributed to adsorbed water emerged at 536.3 eV in the O 1s spectrum of Char_L_ (Figure 2c), but not in that of Char_L/Fe_ or Char_C_. This observation again proves that the Char_L_ surface can absorb water while Char_L/Fe_ and Char_C_ reject water.

**Figure 3:**
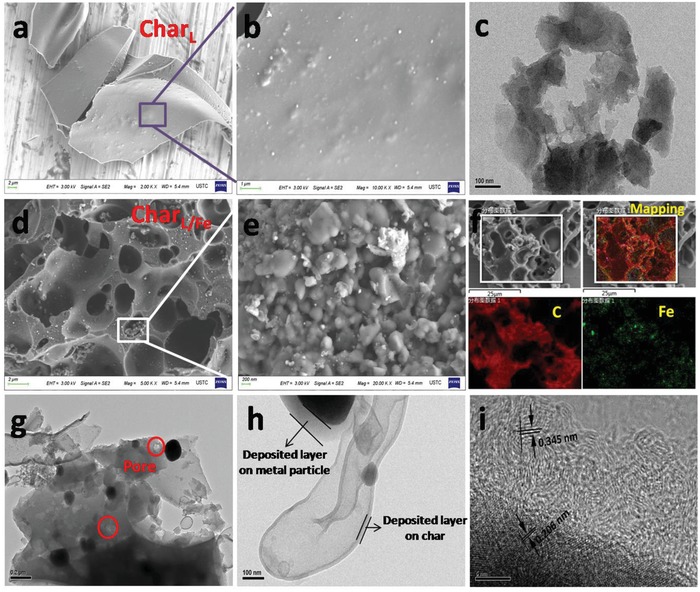
SEM images of a,b) Char_L_, d,e) Char_L/Fe_; f) SEM‐EDS images of Char_L/Fe_; TEM images of Char_L_ (c) and g–i) Char_L/Fe_.

To observe the new formed coverage on Char_L/Fe_ and the microscopic morphology, SEM and TEM imaging were performed. From the images in **Figure** **3**, both Char_L_ and Char_L/Fe_ exist as irregular blocks. The surface of Char_L_ is smooth (Figure 3a,b) while that of Char_L/Fe_ is relatively rough with abundant pores and small particles (Figure 3d,e). It has been well demonstrated that the hydrophobicity can be significantly increased by improving the surface roughness.^[^ 6, 16 ^]^ Thus, the cruder surface of Char_L/Fe_ may contribute to the amplification of its hydrophobicity in comparison with Char_L_. To investigate the distribution of Fe, element mapping images were taken (Figure 3f). The results show that a portion of Fe exists in the form of agglomerated nanoparticles, while the majority dispersedly distributed in the various regions of biochar, contributing to the uniform deposition of a superhydrophobic carbon layer on the surface. Pores and metal particles with size of 100–200 nm can be seen in the Char_L‐Fe_ from the TEM image (Figure 3g), and the edges of both metal particles and char particles are surrounded by new layers with the thickness of about 100 and 30 nm, respectively (Figure 3h). In terms of chrominance, the layers in the two places are similar, implying the same phase. The compositions of the crystal phases were analyzed with HRTEM (Figure 3i). In the darker region, the spacing between neighboring parallel fringes was found to be approximately 0.206 nm, which agrees well with the (101) lattice plane spacing of C_0.09_Fe_1.91_. Furthermore, the spacing of lattice stripes is 0.345 nm in the lighter colored region, which is a typical characteristic of (002) lattice plane spacing of graphite carbon. By comparison, neither pores nor new layers can be found in the TEM image of Char_L_.

**Figure 4:**
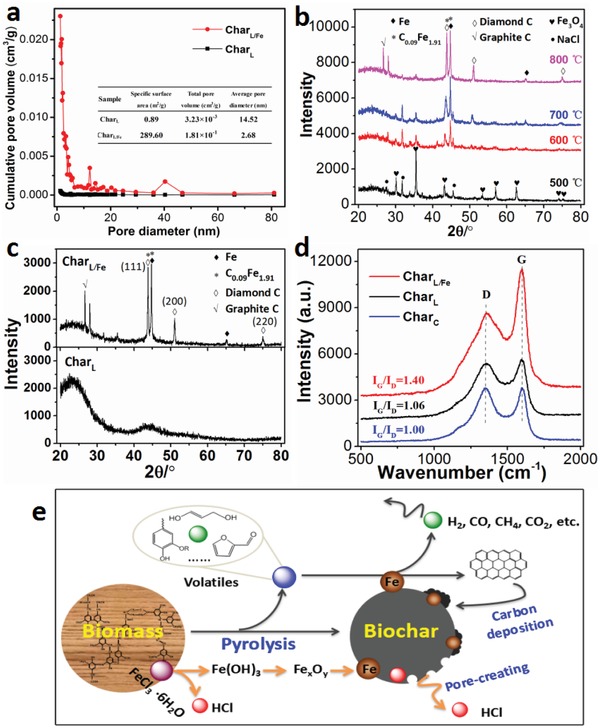
a) The pore size distribution of Char_L_ and Char_L/Fe_, and the insert table lists the data of their pore property; b) XRD spectra (Cu/0.154 nm) of Char_L/Fe_ pyrolyzed at different temperatures; c) XRD spectra (Cu/0.154 nm) of Char_L/Fe_ and Char_L_ pyrolyzed at 800 °C; d) Raman spectra of different chars; e) an illustrative diagram for the catalytic mechanism of FeCl_3_ in the preparation of superhydrophobic biochar.

The addition of some chlorides can catalyze the pore‐creating in the process of biomass pyrolysis, which will substantially improve the porosity of biochar.^[^ 18 ^]^ The pore structures of Char_L_ and Char_L/Fe_ were determined through N_2_ adsorption/desorption. As shown in **Figure** **4**a, the Char_L/Fe_ shows a higher porosity than Char_L_ at all the tested range of pore size. The specific surface area and total pore volume of Char_L/Fe_ is 289.60 m^2^ g^−1^ and 1.81 × 10^−1^ cm^3^ g^−1^, respectively, far larger than the 0.89 m^2^ g^−1^ and 3.23 × 10^−3^ cm^3^ g^−1^ of Char_L_. This result is consistent with the observation from the SEM and TEM images, supporting that the catalytic pore‐creating effect of FeCl_3_ increased the porosity of biochar and consequently enhanced the surface roughness.

XRD was employed to measure the crystalline phase. As shown in Figure 4b, after pyrolysis at 500 °C, the Fe element in Char_L/Fe_ mainly exists as Fe_3_O_4_ (JCPDS NO. 65–3107). As the temperature increased, the peaks of Fe_3_O_4_ gradually weakened as new peaks arose. In the Char_L/Fe_ pyrolyzed at 800 °C, the Fe element was converted into Fe^0^ (JCPDS NO. 06–0696) and martensitic alloy C_0.09_Fe_1.91_ (JCPDS NO. 44–1292). Fe species can be reduced to Fe^0^ by reductive gas and carbon produced in the biomass pyrolysis process.^[^ 22 ^]^ Additionally, the Fe° can melt in hydrocarbon gas, producing iron‐carbon alloy and carbon at high temperature.^[^ 23 ^]^ Moreover, in the XRD spectrum (Figure 4c) of Char_L/Fe_ pyrolyzed at 800 °C, there are obvious peaks belonging to diamond and graphitic carbon. The peaks at 43.8°, 51.1°, and 75.1° can be assigned to the (111), (200), and (220) planes of diamond, respectively (JCPDS NO. 43–1104).^[^ 24 ^]^ Based on the correlative reports, diamond‐like carbon can be prepared by chemical vapor deposition of gaseous hydrocarbons at temperatures under 1000 °C, and it can also be produced via graphite conversion at ultrahigh temperatures above 2000 °C.^[^ 25 ^]^ In this work, it was deemed to be generated through chemical vapor deposition of gaseous hydrocarbons, due to the pyrolysis temperature of 800 °C. Based on the XRD results, the transformation of Fe species in pyrolysis process can be described by Equations (1)–(5).

(1) $${\text{FeCl}}_{\text{3}}\cdot 6{\text{H}}_{2}\text{O}\;\to \;\text{Fe}{\left(\text{OH}\right)}_{3}\;+\;3\text{HCl}$$

(2) $${\text{2Fe(OH)}}_{\text{3}}\;\to \;{\text{Fe}}_{2}{\text{O}}_{3}\;+\;3{\text{H}}_{2}\text{O}$$

(3) $$3{\text{Fe}}_{2}{\text{O}}_{3}\;+\;4{\text{H}}_{2}(\text{CO},\text{C})\;\to \;2{\text{Fe}}_{3}{\text{O}}_{4}\;+\;4{\text{H}}_{2}\text{O}({\text{CO}}_{2},\text{CO})↑$$

(4) $${\text{Fe}}_{3}{\text{O}}_{4}\;+\;2\text{C}\;\to \;3\text{Fe}\;+\;4{\text{CO}}_{2}↑$$

(5) $${\text{C}}_{\text{x}}{\text{H}}_{\text{y}}{\text{O}}_{\text{z}}\;+\;\text{Fe}\to \text{C}\;+\;{\text{C}}_{0.09}{\text{Fe}}_{1.91}\;+\;{\text{H}}_{2}↑\;+\;\text{CO}↑\;+\;{\text{CO}}_{2}↑$$

Fe species can catalyze the deposition reactions of some gaseous hydrocarbons, forming carbon of high graphitization degree.^[^ 17a ^]^ The Raman spectrum was employed to analyze the graphitization degree of the samples. As shown in Figure 4d, the *I*_G_/*I*_D_ value of Char_L/Fe_ is 1.40, much larger than the 1.06 of Char_L_, exhibiting a higher graphitization degree. It supports the foregoing inference that FeCl_3_ promoted the formation of a new carbon layer, which has a high graphitization degree and low defect content, leading to a low surface energy. The compositions of lignin pyrolytic gases with and without FeCl_3_ catalysis were measured sequentially, and the results show that the content of methane and other hydrocarbons in pyrolysis gas decreased, while the percentage of hydrogen increased by 61.9% under the catalysis of FeCl_3_, compared with that without catalysis (Figure S2 and Table S7, Supporting Information). This coincides with the typical process of catalytic deposition of hydrocarbons to form aromatic compounds and release hydrogen.

According to the above experimental and characterization results, the catalytic mechanism of FeCl_3_ in the preparation of superhydrophobic biochar can be summarized as follows (Figure 4e). During the pyrolysis process, biomass decomposed to produce small molecular volatiles (including condensable oils and non‐condensable gases). Meanwhile, the uniformly preloaded FeCl_3_ was hydrolyzed and decomposed to produce iron oxides and HCl. The HCl catalyzed the decomposition of biomass to produce a large number of pores in the residual char, which made the surface of biochar rough. On the other hand, the iron oxides were reduced to zero‐valent iron under the action of reductive gases and carbon at the high temperature. The pyrolysis products of biomass contacted with the zero‐valent iron, and chemical vapor deposition occurred under its catalysis to form hydrophobic carbon and Fe/C alloy. After a period of reaction, a new rough carbon coating containing few polar sites was formed on the surface of biochar, covering the original components and making the biochar superhydrophobic. The formation of superhydrophobic surface is attributed to the synergistic effect of Cl and Fe, and replacing FeCl_3_ with Fe(NO_3_)_3_, Fe_2_(SO_4_)_3_, NiCl_2_, or NaCl cannot achieve the same results (Figure S3, Supporting Information).

### 2.3 Usability and Environmental Stability

**Figure 5:**
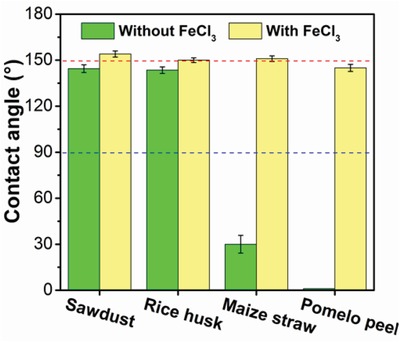
Water contact angle test results of biochars derived from different biomass waste and that catalyzed by FeCl_3_.

Based on the above catalytic mechanism, it can be speculated that this method can be applied to biomass with different components. Several typical lignocellulosic biomasses (i.e., sawdust, rice husk, corn straw, and pomelo peel) were used to verify the usability of this method. The results of water contact angle tests showed that the sawdust char and rice husk char were hydrophobic, whereas the corn straw char and pomelo peel char were hydrophilic and superhydrophilic, respectively (**Figure** **5**). Under the catalysis of FeCl_3_, the sawdust, rice husk, and maize straw‐derived chars attained superhydrophobicity, with contact angle above 150°, and pomelo peel‐derived char reached near‐superhydrophobicity. Pyrolysis in the presence of FeCl_3_ was proven to be an effective method to prepare superhydrophobic biochar materials, even there are a lot of hydrophilic ashes existed (Table S8, Supporting Information).

### 2.4 Environmental Stability and Applications

**Figure 6:**
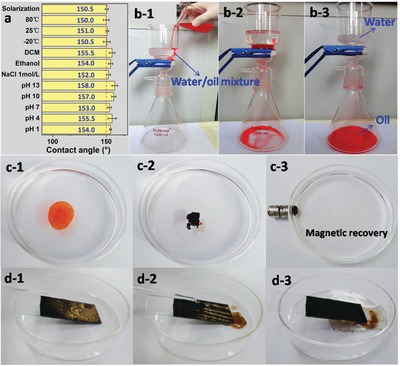
a) Water contact angles of Char_L/Fe_ after aging at different conditions; b‐1,2,3) pictures of oil/water separation process with Char_L/Fe_‐loaded filtration equipment; c‐1,2,3) pictures of the selective oil adsorption with Char_L/Fe_ and the magnetic recovery of oil and carbon material; d‐1,2,3) pictures of the self‐cleaning process on the Char_L/Fe_ surface.

To meet the practical requirements, the superhydrophobic material was required to possess good stability in various environmental conditions. The superhydrophobic stability of biochar was validated after aging in different pH, temperature, ionic strength, solvent, and solarization conditions for 15 d. The results reveal that the superhydrophobic property of biochar did not diminish, but improved after treatment in aqueous solutions with pH varying from 1–13 (**Figure** **6**a). Especially after aging in aqueous alkali with a pH of 13, the water contact angle of obtained biochar increased to 158.0° from the initial 151.5°. The water contact angle of Char_L/Fe_ treated at pH of 1, 4, 7, 10 is 154.0°, 155.5°, 153.0°, 157.0°, respectively, suggesting the excellent acid–base tolerance of the material. In addition, the superhydrophobic property of biochar aged in NaCl solution, ethanol, and dichloromethane was also enhanced with water contact angle of 152.0°, 154.0°, 155.5°, respectively. It is mainly because that the ash and soluble polar compounds in biochar were washed out during the wet‐aging process.^[^ 26 ^]^ The water contact angles of samples aged at −20, 25, 80 °C, and under solarization are 150.5°, 151.0°, 150.0°, and 150.5°, respectively, slightly smaller than the original 151.5°. In summary, the superhydrophobic biochar prepared in this study maintains excellent stability against harsh circumstances.

The superhydrophobic materials are widely used in environment remediation and pollutants separation.^[^ 27 ^]^ For instance, superhydrophobic materials can be used for oil/water separation. To achieve this application, the Char_L/Fe_ was bonded to a stainless steel sieve as described in Figure S4, Supporting Information. The oil/water mixture (30%, v/v) was poured into the filtration equipment, and the separation was achieved by the gravity of the liquids. During the separation process (Figure 6b), water contacted the mesh earlier but could not pass through, while the subsequent oil droplet permeated through the Char_L/Fe_ coated mesh surface and entirely flowed into the conical flask without any assistance from external forces. Finally, the sudan red dyed oil and water were separated successfully. This equipment can separate oil with density larger than water, but most oil will float on the water and cannot be separated with this mesh. However, the excellent superhydrophobic and superoleophilic properties of Char_L/Fe_ would be promising to solve this separation problem by directly and selectively absorbing oil on the water surface. As shown in Figure 6c, sudan red dyed cyclohexane was dropped into water. When the Char_L/Fe_ material was added, the oil was rapidly adsorbed. Taking advantage of its magnetic response property, the Char_L/Fe_, together with oil, can be separated from water with the assistance of a magnet (Figure 6c‐3). Additionally, the superhydrophobic Char_L/Fe_ can be used to build a self‐cleaning surface. When water droplets were dropped onto the contaminated surface, the soil powder was immediately picked up, surrounded, and carried away by the rolling water droplets, leaving behind a clean surface (Figure 6d). The water droplets maintained a spherical shape and left no remnant on the surface.

## 3 Conclusion

In summary, because of the presence of polar atoms such as O, N, P, S, and metals, most of the biochar is hydrophilic or weakly hydrophobic, which limits the value‐added application of biochar. In this work, superhydrophobic biochar was successfully prepared by using the catalytic effect of FeCl_3_ in carbon deposition and pore‐creating during the pyrolysis of biomass. Currently study cannot only dispose the biomass waste, but also effective prepare novel functional carbon materials. The process was simple, cheap, environmental friendly, and showed an outstanding universality for different lignocellulosic biomasses, which will definitely expand the application of biochar and become an ideal substitute for graphene and carbon nanotubes.

## 4 Experimental Section

##### Materials

FeCl_3_·6H_2_O (AR), NaCl (AR), HCl (AR), NaOH (AR), ethanol (AR), dichloromethane (DCM, AR), and microcrystalline cellulose were purchased from Sinopharm Chemical Reagent Co., Shanghai, China. Lignin was purchased from Nantong, China. All the biomass (sawdust, rice husk, pomelo peel, maize straw) used in this work was pulverized into powders with a high‐speed rotary grinder, and then the particles with size between 0.15 and 0.25 mm (60–100 mesh) were collected and dried at 105 °C overnight for further use. Ultrapure water was used in all experiments.

##### Preparation of Biochar and Superhydrophobic Biochar

To prepare biochar, the sample precursor was fast pyrolyzed at a certain temperature and the obtained biochar was held for additional 30 min, as reported in our previous works.^[^ 28 ^]^ To obtain the superhydrophobic biochar, in a typical run, 10 g biomass sample was dispersed in 200 mL of 0.05 mol L^−1^ FeCl_3_ solution to achieve a doping ratio of 1 mmol g^−1^ and magnetically stirred for 24 h, followed by dehydration with a rotary evaporator at 60 °C. Thereafter, the solid was transferred into a culture dish and dried at 105 °C in a drier. 12 h later, the sample was uniformly ground to near the size of the original sawdust (0.15–0.25 mm), obtaining the FeCl_3_ preloaded biomass. Afterward, the composite was pyrolyzed to obtain superhydrophobic biochar. The final products were denoted as follows: the unmodified lignin char was marked as Char_L_‐t; the superhydrophobically modified lignin char was denoted as Char_L/Fe_‐t; and the cellulose char was abbreviated as Char_C_‐t. The t stands for the pyrolysis temperature (*t* = 500, 600, 700, or 800 °C), which is defaulted as 800 °C without special explanation.

##### Water Contact Angle Determination

The wetting properties of biochars were measured by testing the water contact angle on a static contact angle measuring instrument (JC2000C1, Zhongchen Digital Technic Apparatus Co., Ltd., Shanghai, China). In a typical run, the biochar powder was uniformly spread on a glass slide with the assistance of double‐sided tape.^[^ 2 ^]^ To measure the water contact angle of the sample, 2 µL of pure water droplet was dropped onto the sample surface and photographed at room temperature. Each sample was repeated three times, and the average value was recorded.

##### Surface Energy Measurement

Surface energy (γ_s_) consists of two components: dipole‐hydrogen bonding (γ_s_^p^) and dispersion (γ_s_^d^) forces (i.e., γ_s_ = γ_s_^d^ + γ_s_^p^), which can be calculated with Equations (6)–(8) according to the Owens–Wendt method^[^ 19 ^]^ using the experimentally determined intrinsic contact angles (θ_1_ and θ_2_) of water (γ_w_ = 72.8 mN m^−1^, γ_w_^p^ = 51 mN m^−1^, γ_w_^d^ = 21.8 mN m^−1^) and hexadecane (γ_h_ = 27.6 mN m^−1^, γ_h_^p^ = 0 mN m^−1^, γ_h_^d^ = 27.6 mN m^−1^):

(6) $$\gamma_{\text{w}}(1\;+\;\cos\theta_{1})\;=\;2{\left(\gamma_{\text{s}}^{\text{d}}\gamma_{\text{w}}^{\text{d}}\right)}^{1/2}\;+\;2{\left(\gamma_{\text{s}}^{\text{p}}\gamma_{\text{w}}^{\text{p}}\right)}^{1/2}$$

(7) $$\gamma_{\text{h}}(1\;+\;\cos\theta_{2})\;=\;2{\left(\gamma_{\text{s}}^{\text{d}}\gamma_{\text{h}}^{\text{d}}\right)}^{1\text{/}2}\;+\;2{\left(\gamma_{\text{s}}^{\text{p}}\gamma_{\text{h}}^{\text{p}}\right)}^{1\text{/}2}$$

(8) $$\gamma_{\text{s}}=\gamma_{\text{s}}^{\text{d}}\;+\;\gamma_{\text{s}}^{\text{p}}$$

##### Test of Stability

Aiming to investigate the stability of the superhydrophobic property of biochar under harsh circumstances, the superhydrophobic biochar was aged under various conditions, such as different pH, temperature, ionic strength, solvent, and solarization. In a typical run, the biochar powder was immersed into aqueous solution or organic solvent, in an airtight 100 mL conical flask, and kept for 15 days at ambient temperature. Before the contact angle test, the biochar was separated from the suspension via centrifugation and dried at 50 °C in a vacuum drying oven. HCl and NaOH were used to adjust the pH value of the aqueous solution, and NaCl was used to supply ionic strength. In the investigation of temperature and solarization, the biochar was placed into a refrigerator (−20 °C), an incubator (25 °C), an oven (80 °C), and outdoors, sequentially, for 15 days before the water contact angle was tested.

##### Characterizations

The functional groups of samples were investigated by employing Fourier transform infrared spectroscopy (FTIR, EQUIVOX55 IR spectroscopy, Bruker, Germany) with detection range varying from 4000 to 400 cm^−1^. X‐ray diffraction (XRD) patterns were acquired using an 18 kW rotating anode X‐ray diffractometer (MXPAHF, Rigaku, Japan), with a nickel‐filtered Cu Kα radiation source (30 kV/160 mA, λ = 0.154 nm), scanning from 20° to 80° at a scan rate (2θ) of 0.02° s^−1^. The diffraction peaks were analyzed based on the powder diffraction file database using the XRD data analysis software (MDI JADE 5.0). A scanning electron microscope (SEM, Sirion 200, FEI Electron Optics Company, USA) was used to observe the morphology of the samples. SEM‐EDS images of the samples were obtained by energy dispersive spectroscopy (EDS, INCA energy, UK). High resolution transmission electron microscope (HRTEM, JEM‐2100F, JEOL, Japan) was used to analyze the morphology of samples and crystal lattice structures. The composition of ash was determined by X‐ray fluorescence spectrometry (XRF, XRF‐1800, SHIMADZU Co., Japan). XPS spectra were recorded with an X‐ray photoelectron spectrometer (ESCALAB250, Thermo‐VG Scientific, UK). Elemental (C, H, N, S) analyses were conducted using an elemental analyzer (Vario EL Cube, Elementar, Germany). The contents of Fe and Na were measured using an atomic absorption spectrophotometer (AAS‐4530F, Shanghai Precision and Scientific Instrument Co., Ltd., China) after the solid samples were digested. Oxygen content was determined by mass balance. Pore structures of samples were measured by nitrogen adsorption–desorption isotherm experiments at 77 K in a Micromeritics Gemini apparatus (Tristar II 3020M, Micromeritics Instrument Co., USA). The Brunauer–Emmett–Teller (BET) method was used to calculate specific surface areas of the samples, and Barrett–Joyner–Halenda (BJH) method was adopted to calculate their pore volumes. The Raman analysis was performed with a Laser Raman spectrometer (LabRamHR, HORIBA Jobin Yvon, France). The wavelength of radiation source was 514 nm.

## Conflict of Interest

The authors declare no conflict of interest.

## Supporting information

Supporting Information
