# Supplementary material for: One‐Step Thermochemical Conversion of Biomass Waste into Superhydrophobic Carbon Material by Catalytic Pyrolysis
Source: Glob Chall. 2020 Feb 20;4(4):1900085. doi: 10.1002/gch2.201900085 (PMC7117845; doi:10.1002/gch2.201900085)
Supplement: Supplementary file 1 — Supporting Information [file GCH2-4-1900085-s001.pdf]

Copyright WILEY-VCH Verlag GmbH & Co. KGaA, 69469 Weinheim, Germany, 2020.

Global Challenges
Open Access

Supporting Information

for *Global Challenges,* DOI: 10.1002/gch2.201900085

One-Step Thermochemical Conversion of Biomass Waste into Superhydrophobic Carbon Material by Catalytic Pyrolysis

*De-Chang Li, Wan-Fei Xu, Hui-Yuan Cheng, Kun-Fang Xi, Bu-De Xu, and Hong Jiang**

One-Step Thermochemically Converting Biomass Waste into Superhydrophobic Carbon Material by Catalytic Pyrolysis

De-Chang Li, Wan-Fei Xu, Hui-Yuan Cheng, Kun-Fang Xi, Bu-De Xu, and Hong Jiang *

* CAS Key Laboratory of Urban Pollutants Conversion, Department of Applied Chemistry, University of Science and Technology of China, Hefei 230026, China.

Corresponding Author's Email Address: jhong@ustc.edu.cn

1.Tables

Table S1. Comparisons of literatures reporting preparation of hydrophobic carbon materials from biomass.

Table S2. Ash contents of different samples.

Table S3. The water contact angle results of different biochar samples.

Table S4. The significant difference analysis between different biochar samples based on the water contact angle results in Table S1.

Table S5. Results of surface energy test.

Table S6. XPS C1s and O1s results of $Char_L$ and $Char_{L/Fe}$.

Table S7. The components of pyrolysis gas of lignin and $FeCl_3$/lignin, and data analysis.

Table S8. Ash contents of different samples.

2. Figures

Figure S1 Typical images of water contact angle test of different samples.

Figure S2 Volume percent content of main gaseous products from lignin pyrolysis with and without $FeCl_3$ catalysis.

Figure S3. Water contact angle result of lignin char catalyzed by different compounds.

Figure S4. Pictures of stainless steel sieve before (a) and after (b) $Char_{L/Fe}$ coating.

3. References

1. Tables

Table S1. Comparisons of literatures reporting preparation of hydrophobic carbon materials from biomass.

No.	Precursor	Method	Heat treatment condition	Water CA (^{o})	Ref.
1	Nanocellulose	Freezing–drying and TiO_2 coating	---	>90	(1)
2	Cotton	Carbonization with $FeCl_3$	5 ^{o}C/min, 800 ^{o}C for 3 h	132.8	(2)
3	Cotton	Carbonization	5 ^{o}C/min, 850 ^{o}C for 2 h	>90	(3)
4	Winter melon	Hydrothermal treatment, freezing–drying, carbonization	180 ^{o}C for 10 h, 800 ^{o}C for 1 h,	135	(4)
5	Glucose	Hydrothermal treatment, freeze-drying, polydimethylsiloxane coating	180 ^{o}C for 12-48 h	158	(5)
6	Waste newspaper	Freezing–drying, post-pyrolysis	2 ^{o}C/min, 400 ^{o}C for 1 h; 5 ^{o}C/min, 900 ^{o}C for 2 h	132	(6)
7	Cellulose	Freezing–drying, post-pyrolysis	5 ^{o}C/min, 900 ^{o}C for 15 min	149	(7)
8	Lignin and polymer	Polymerization, post-pyrolysis	5 ^{o}C/min, 700 ^{o}C for 2 h	127.2	(8)
9	Waste paper	Freezing–drying, post-pyrolysis	5 ^{o}C/min, 850 ^{o}C for 2 h	>90	(9)
10	Lignocellulosic biomass	Fast pyrolysis with $FeCl_3$	>200 ^{o}C/min, 800 ^{o}C for 0.5 h	~150	**This study**

Table S2. Ash contents of different samples.

Sample	Ash content (%)
Crude lignin	3.71± 0.05
$Char_L$-500	5.93± 0.14
$Char_L$-600	7.02± 0.11
$Char_L$-700	7.95± 0.20
$Char_L$-800	9.88± 0.25

Table S3. The water contact angle results of different biochar samples.

No.	Sample	Test 1	Test 2	Test 3	Average	Standard deviation
1	$Char_{L}$-500	86.5	90.5	96.0	91.0	4.77
2	$Char_{L}$-600	~0	~0	~0	~0	0
3	$Char_{L}$-700	~0	~0	~0	~0	0
4	$Char_{L}$-800	~0	~0	~0	~0	0
5	$Char_{L/Fe}$-500	120.5	127.0	124.5	124.0	3.28
6	$Char_{L/Fe}$-600	148.5	145.0	146.0	146.5	1.80
7	$Char_{L/Fe}$-700	150.5	149.0	147.5	149.0	1.50
8	$Char_{L/Fe}$-800	152.5	150.5	151.5	151.5	1.00

Table S4. The significant difference analysis between different biochar samples based on the water contact angle results in Table S1.

**No.**	Sample 1	Sample 2	P value	Result
1	$Char_L$-500	$Char_{L/Fe}$-500	0.0006	Significant
2	$Char_L$-600	$Char_{L/Fe}$-600	<0.0001	Significant
3	$Char_L$-700	$Char_{L/Fe}$-700	<0.0001	Significant
4	$Char_L$-800	$Char_{L/Fe}$-800	<0.0001	Significant

Table S5. Results of surface energy test.

Sample	Surface energy (mN/m)		
	γ_s	γ_s^d	γ_s^p
**$Char_L$**	73.29	27.60	45.69
**$Char_{L/Fe}$**	19.25	11.32	7.94

Table S6. XPS C1s and O1s results of $Char_L$ and $Char_{L/Fe}$.

Sample	Position (eV)	Attribution	Percentage (%)	Position (eV)	Attribution	Percentage (%)
**$Char_L$**	284.28	C＝C	16.8	530.97	C＝O in quinones	9.2
	284.67	Graphitic C	28.2	531.71	C＝O in ketones, aldehydes	18.6
	285.10	C—H	34.7	532.49	C—OH	28.7
	285.76	C—C or C—O—C	20.4	533.47	C—O—C	37.6
				536.32	Adsorbed water	6.0
**$Char_{L/Fe}$**	284.18	C＝C	7.1	530.48	C＝O in quinones	9.1
	284.57	Graphitic C	30.1	531.50	C＝O in ketones, aldehydes	14.4
	284.98	C—H	39.4	532.42	C—OH	29.1
	285.51	C—C	23.4	533.45	C—O—C	47.5

Table S7. The components of pyrolysis gas of lignin and $FeCl_3$/lignin, and data analysis.

Gas	Lignin		$FeCl_3$/lignin		Lignin		$FeCl_3$/lignin		P value of T-test
	Test 1	Test 2	Test 1	Test 2	Average	Standard deviation	Average	Standard deviation	
**H_2(%)**	23.796	23.546	38.601	38.047	23.67	0.18	38.32	0.39	**0.0004**
**CO(%)**	30.040	30.860	27.150	27.172	30.45	0.58	27.16	0.02	**0.0152**
**CO_2(%)**	16.001	17.401	15.238	15.036	16.70	0.99	15.14	0.14	**0.1575**
**CH_4(%)**	23.184	23.562	15.901	15.583	23.37	0.27	15.74	0.22	**0.0010**
**Others(%)**	6.979	4.631	3.110	4.162	5.81	1.66	3.64	0.74	**0.2338**

Table S8. Ash contents of different samples.

Sample	Ash content (%)	Sample	Ash content (%)
Cellulose	< 0.01	Crude lignin	3.71± 0.05
Sawdust char	4.36± 0.12	$Char_L$-F500	5.93± 0.14
Rice husk char	37.23± 0.34	$Char_L$-F600	7.02± 0.11
Maize straw char	17.91± 0.46	$Char_L$-F700	7.95± 0.20
Pomelo peel char	13.51± 0.15	$Char_L$-F800	9.88± 0.25
$Char_C$	0.02	$Char_{L/Fe}$	10.28± 0.34

2. Figures

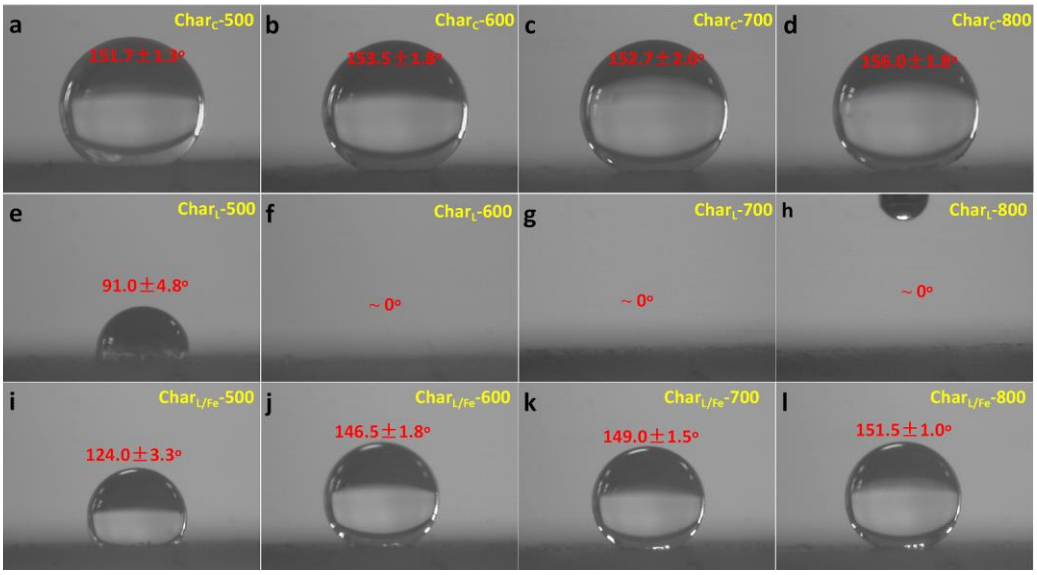

Figure S1. Typical images of water contact angle test of different samples.

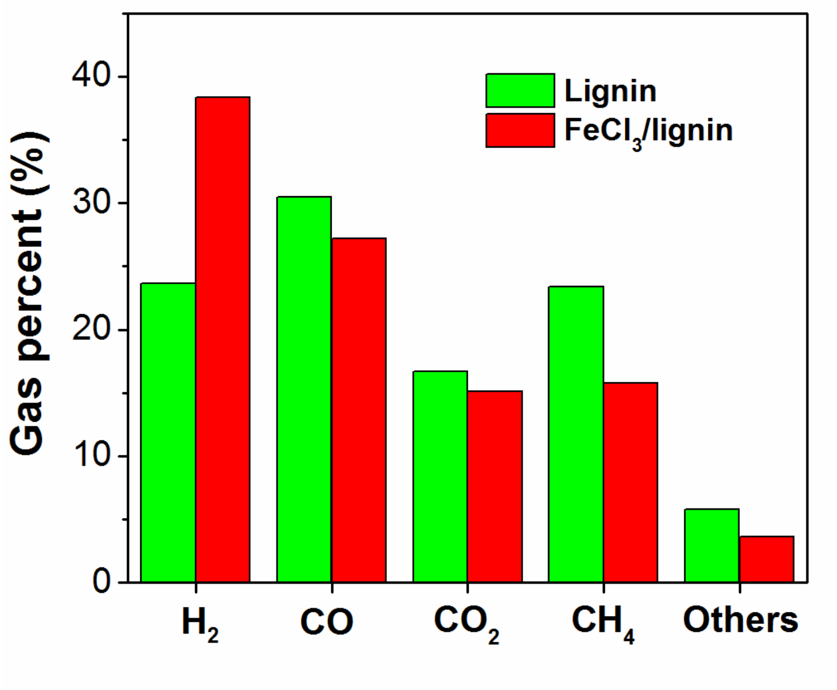

Figure S2. Volume percent content of main gaseous products from lignin pyrolysis with and without $FeCl_3$ catalysis.

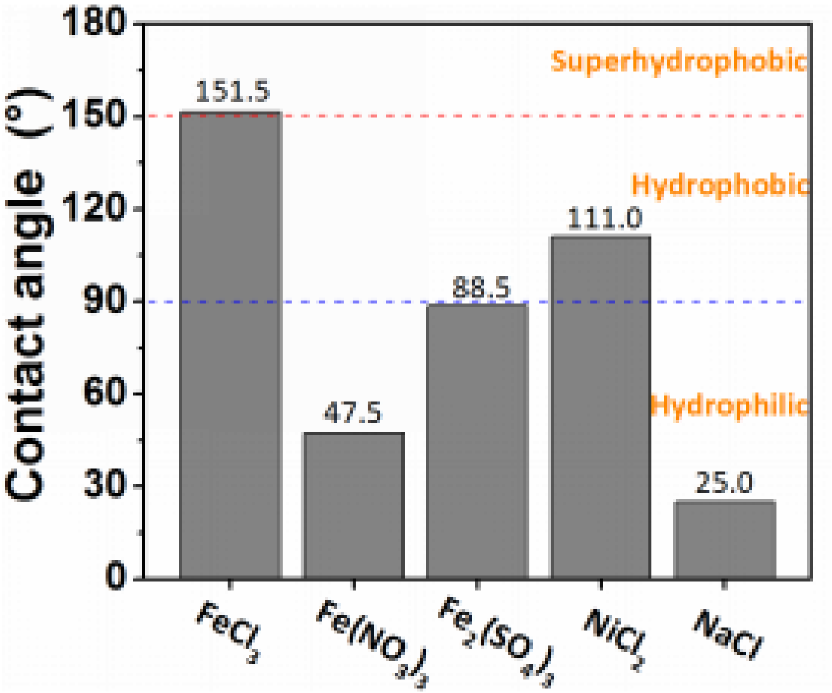

Figure S3. Water contact angle result of lignin char catalyzed by different compounds.

To investigate the roles of Cl and Fe in the hydrophobic modification process, a series of control experiments were conducted. A certain amount of $Fe(NO_3)_3$, $Fe_2(SO_4)_3$, $NiCl_2$, or NaCl was respectively used to replace $FeCl_3$ during the preparation process, holding either Cl or Fe at the same concentration with that in $FeCl_3$. The wettability of obtained materials was determined and shown in Figure S3. The char samples modified with NaCl, $Fe(NO_3)_3$ and $Fe_2(SO_4)_3$ are all hydrophilic, with the contact angle below 90°, while that modified with $NiCl_2$ is hydrophobic. It indicates that not all chloride and ferric salts can catalyze the hydrophobic modification of $Char_L$. Thus, the effect is not resulted from the alone existence of either Cl or Fe, but attributed to the combined actions of Cl and Fe.

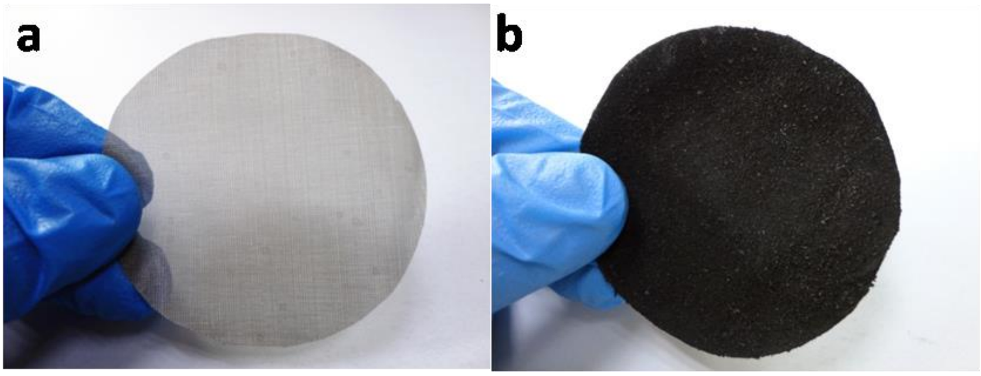

Figure S4. Pictures of stainless steel sieve before (a) and after (b) $Char_{L/Fe}$ coating.

To achieve the application of oil/water separation, the $Char_{L/Fe}$ was bonded to a stainless steel sieve with adhesive which was insoluble in organic solvent. Briefly, a piece of stainless steel sieve with pore density of 300 meshes was cut into a circular shape, based on a filter membrane (Fig. S9a), and washed with ethanol for further use. The glue was prepared by dissolving 2 g of resin E-44 and low molecular polyamide resin into 5 mL of dichloromethane. After the glue was daubed onto the mesh for several minutes, the $Char_{L/Fe}$ powder was uniformly planted on it and aged for 24 h at room temperature, obtaining the $Char_{L/Fe}$ coated mesh as shown in Fig. S9b. Then, the mesh was assembled in filtration equipment. Dichloromethane, whose density is larger than water, was chosen as testing oil and dyed with sudan red. The oil/water mixture (30 v/v%) was poured into the filtration equipment, and the separation was achieved by the gravity of the liquids.

3. References

(1) Korhonen, J. T.; Kettunen, M.; Ras, R. H. A.; Ikkala, O. Hydrophobic nanocellulose aerogels as floating, sustainable, reusable, and recyclable oil absorbents. *Acs Appl. Mater. Inter.* **2011,** *3* (6), 1813-1816.

(2) Liu, R.-L.; Li, X.-Q.; Liu, H.-Q.; Luo, Z.-M.; Ma, J.; Zhang, Z.-Q.; Fu, Q. Eco-friendly fabrication of sponge-like magnetically carbonaceous fiber aerogel for high-efficiency oil-water separation. *Rsc Adv.* **2016,** *6* (36), 30301-30310.

(3) Bi, H.; Yin, Z.; Cao, X.; Xie, X.; Tan, C.; Huang, X.; Chen, B.; Chen, F.; Yang, Q.; Bu, X.; Lu, X.; Sun, L.; Zhang, H., Carbon fiber aerogel made from raw cotton: A novel, efficient and recyclable sorbent for oils and organic solvents. *Adv. Mater.* **2013,** *25* (41), 5916-5921.

(4) Li, Y.-Q.; Samad, Y. A.; Polychronopoulou, K.; Alhassan, S. M.; Liao, K. Carbon aerogel from winter melon for highly efficient and recyclable oils and organic solvents absorption. *ACS Sustain. Chem. Eng.* **2014,** *2* (6), 1492-1497.

(5) Liang, H.-W.; Guan, Q.-F.; Chen, L.-F.; Zhu, Z.; Zhang, W.-J.; Yu, S.-H. Macroscopic-scale template synthesis of robust carbonaceous nanofiber hydrogels and aerogels and their applications. *Angew. Chem. Int. Edi.* **2012,** *51* (21), 5101-5105.

(6) Han, S.; Sun, Q.; Zheng, H.; Li, J.; Jin, C. Green and facile fabrication of carbon aerogels from cellulose-based waste newspaper for solving organic pollution. *Carbohyd. Polym.* **2016,** *136*, 95-100.

(7) Meng, Y.; Young, T. M.; Liu, P.; Contescu, C. I.; Huang, B.; Wang, S. Ultralight carbon aerogel from nanocellulose as a highly selective oil absorption material. *Cellulose* **2015,** *22* (1), 435-447.

(8) Yang, Y.; Tong, Z.; Ngai, T.; Wang, C. Nitrogen-rich and fire-resistant carbon aerogels for the removal of oil contaminants from water. *Acs Appl. Mater. Inter.* **2014,** *6* (9), 6351-6360.

(9) Bi, H.; Huang, X.; Wu, X.; Cao, X.; Tan, C.; Yin, Z.; Lu, X.; Sun, L.; Zhang, H. Carbon microbelt aerogel prepared by waste paper: An efficient and recyclable sorbent for oils and organic solvents. *Small* **2014,** *10* (17), 3544-3550.
